# Two Novel Hepatocellular Carcinoma Cycle Inhibitory Cyclodepsipeptides from a Hydrothermal Vent Crab-Associated Fungus *Aspergillus clavatus* C2WU

**DOI:** 10.3390/md11124761

**Published:** 2013-12-02

**Authors:** Wei Jiang, Panpan Ye, Chen-Tung Arthur Chen, Kuiwu Wang, Pengyuan Liu, Shan He, Xiaodan Wu, Lishe Gan, Ying Ye, Bin Wu

**Affiliations:** 1 Ocean College, Zhejiang University, Hangzhou 310058, China; E-Mails: jw6912@zju.edu.cn (W.J.); fxzhxx@zju.edu.cn (X.W.); lsgan@zju.edu.cn (L.G.); gsyeying@zju.edu.cn (Y.Y.); 2 Eye center, the second affiliated hospital, Zhejiang University School of Medicine, Hangzhou 310000, China; E-Mail: yepanpan@hotmail.com; 3 Institute of Marine Geology and Chemistry, National Sun Yat-sen University, Kaohsiung 80424, Taiwan; E-Mail: ctchen@faculty.nsysu.edu.tw; 4 Department of Applied Chemistry, Zhejiang Gongshang University, Hangzhou 310058, China; E-Mail: wkwnpc@zjgsu.edu.cn; 5 Center for Intelligent Chemical Instrumentation, Department of Chemistry and Biochemistry, Clippinger Laboratories, Ohio University, Athens, Ohio 45701, OH, USA; E-Mail: pl155809@ohio.edu; 6 Key Laboratory of Applied Marine Biotechnology, Ningbo University, Ministry of Education, Ningbo 315211, China; E-Mail: heshan@nbu.edu.cn

**Keywords:** cyclodepsipeptide, *Aspergillus clavatus*, hydrothermal vent, carcinoma cycle inhibitory

## Abstract

Two novel cyclodepsipeptides containing an unusual anthranilic acid dimer and a d-phenyllactic acid residues, clavatustides A (**1**) and B (**2**), were identified from cultured mycelia and broth of *Aspergillus clavatus* C2WU isolated from *Xenograpsus testudinatus*, which lives at extreme, toxic habitat around the sulphur-rich hydrothermal vents in Taiwan Kueishantao. This is the first example of cyclopeptides containing an anthranilic acid dimer in natural products, and the first report of microbial secondary metabolites from the hydrothermal vent crab. Clavatustides A (**1**) and B (**2**) suppressed the proliferation of hepatocellular carcinoma (HCC) cell lines (HepG2, SMMC-7721 and Bel-7402) in a dose-dependent manner, and induced an accumulation of HepG2 cells in G1 phase and reduction of cells in S phase.

## 1. Introduction

**Chart 1:**
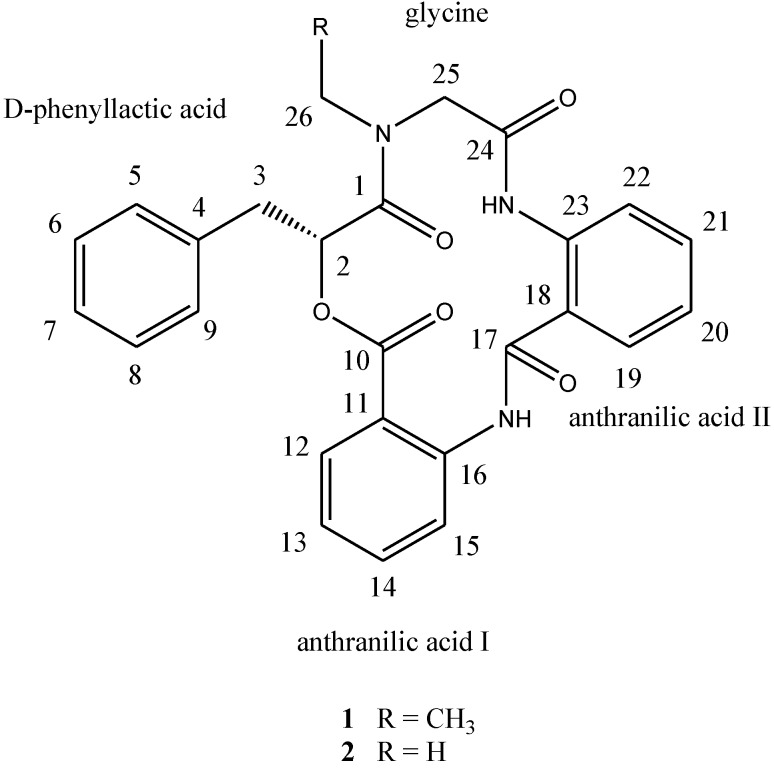
Structures of compounds **1** and **2**.

Marine-derived fungi, living in a stressful habitat, are of great interest as new promising sources of biologically active products. Since marine organisms live in a biologically competitive environment with unique conditions of pH, temperature, pressure, oxygen, light, nutrients and salinity, the chemical diversity of the secondary metabolites from marine fungi is considerably high [1,2,3]. Since ocean hydrothermal vents are among the most dynamic environments on Earth, secondary metabolite diversity of the extreme microbes is considerably high [4,5]. The reports of the loihichelins and the ammonificins were the first publications on new natural products from ocean hydrotermal vent environments [6]. With the advance of sample collecting techniques, ocean hydrothermal vents might be potential hot spots for natural products discovery. Although the occurrence of microbial epibionts on marine crustaceans has been documented, the ecological context is still not fully understood [7,8]. Only a few marine fungi isolates from crab have been chemically and pharmacologically investigated. Phomactin A, which was isolated from the culture filtrate of *Phoma* sp. (SANK 11486), a parasitic marine fungus separated from the shell of a crab *Chinoecetes opilio* by Sugano *et al.* in 1991, showed potent PAF antagonistic activity [9]. Because crab shells not only act as a solid support for surface-populating organisms, but as a source of activating factors and special nutrient components, marine bacteria and fungi isolated from crab are difficult to grow well on terrestrial nutrient broths [8]. The crab *Xenograpsus testudinatus* lives at extreme, toxic habitat around the sulphur-rich hydrothermal vents found in Taiwan Kueishantao [10]. So far, no study of secondary metabolites from a *X. testudinatus* associated fungus has been reported. In order to find drug candidates from the microorganisms which live at extreme and toxic habitat, natural product-producing fungi from the hydrothermal vent crab were cultured in our laboratory. In continuation of our search for anti-cancer natural products, secondary metabolites in the cultured mycelia and broth of *Aspergillus clavatus* C2WU isolated from *X. testudinatus* were investigated. Two novel cyclopeptides containing an unusual anthranilic acid dimer and a d-phenyllactic acid residues, clavatustides A (**1**) and B (**2**) (Chart 1), were identified from cultured mycelia and broth of *A. clavatus* C2WU. Cyclopeptides with an anthranilic acid unit are rare in nature. Two antifungal cyclopeptides with an anthranilic acid unit, sclerotides A and B, were isolated from the marine-derived *Aspergillus sclerotiorum* PT06-1 in a nutrient-limited hypersaline cultural medium [11]. Anthranilic acid dimers with micromolar affinity for the CCK1 receptors were considered to be an important molecular scaffold in medicinal chemistry [12]. To the best of our knowledge, this is the first example of cyclopeptides containing an anthranilic acid dimer in natural products, and the microbial secondary metabolites from the hydrothermal vent crab were rarely reported. Clavatustides A (**1**) and B (**2**) suppressed the proliferation in hepatocellular carcinoma (HCC) cell lines (HepG2, SMMC-7721 and Bel-7402) in a dose-dependent manner, and induced an accumulation of HepG2 cells in G1 phase and reduction of cells in S phase.

## 2. Results and Discussion

The *Aspergillus clavatus* C2WU separated from hydrothermal vent crab *Xenograpsus testudinatus* was cultured. The mycelia and broth were isolated and purified by normal phase column chromatography and reverse phase preparative HPLC.

**Figure 1:**
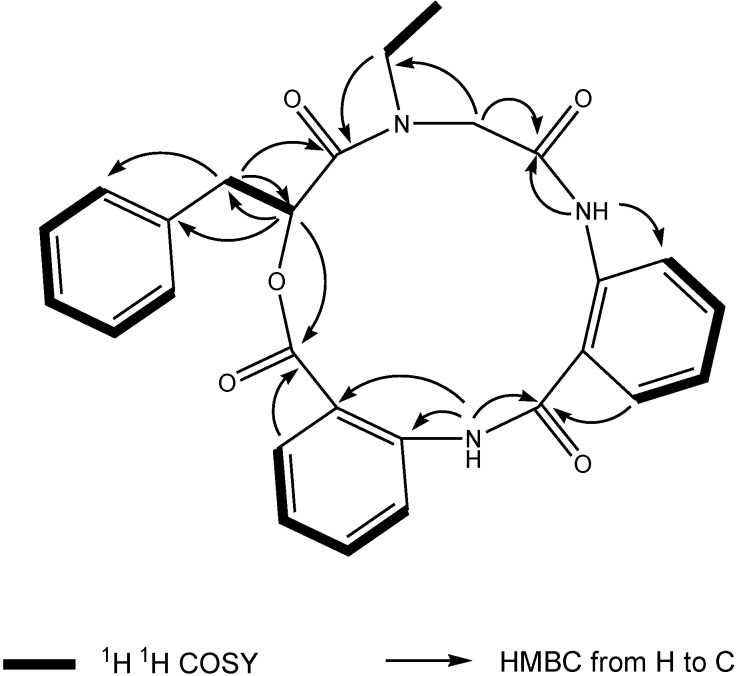
Key ^1^H ^1^H COSY and HMBC correlations of compound **1**.

**Figure 2:**
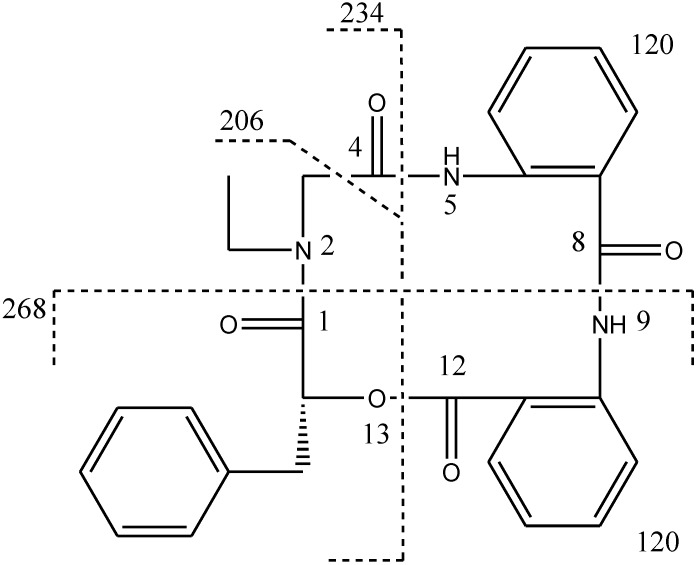
CID illustration of compound **1**.

Compound **1**, [α]^24^_D_ +22 (*c* 0.10, MeOH), UV (MeOH) λ_max_ (log є) 287 (3.11) nm, was obtained as a yellow amorphous solid. The HR-TOF-MS exhibited an ion peak at *m/z* 472.1871 [M + H]^+^ (calcd. for C_27_H_26_N_3_O_5_, 472.1872, corresponding to the molecular formula, C_27_H_26_N_3_O_5_, requiring 17 degrees of unsaturation. The IR spectrum of **1** showed absorption bands at 1726 and 1656 cm^−1^ indicating the presence of both ester and amide functionalities, respectively. The ^13^C NMR spectrum of **1** showed the presence of four carbonyl signals (δ_C_ 169.6, 168.1, 167.3 and 167.1). However, only two amide protons (δ_H_ 8.07 and 10.23) were observed in the ^1^H NMR spectrum. A typical *N*-ethyl carbon resonances at δ_C_ 43.3 and 13.7 in the ^13^C NMR spectrum indicated that **1** possesses an ethylated amide unit. From the above information, compound **1** was deduced to consist of three amide units and one ester unit. In order to elucidate four substructures, a combination of 2D HSQC, HMBC, and ^1^H ^1^H COSY experiments were performed on compound **1** (Figure 1). The HMBC cross peaks from the benzylic methylene protons at δ_H_ 3.46 and 3.25 to the monosubstituted benzene carbons at δ_C_ 129.7, the carbonyl at δ_C_ 169.6 and the oxygenated methine at δ_C_ 71.7 revealed a phenyllactic acid moiety in **1**. Two sequence of four aromatic protons was deduced from ^1^H ^1^H COSY analyses, indicating two *ortho* disubstituted benzenes in **1**. HMBC correlations from doublet protons of disubstituted benzenes to carbonyls and from two amide proton to aromatic carbons of disubstituted benzenes allowed the deduction of two anthranilic acid moieties in **1**. The substructure of *N*-ethyl glycine was inferred from the HBMC correlations from aminated methylene protons at δ_H_ 5.24 and 3.29 to *N*-ethyl carbon at δ_C_ 43.3 and carbonyl at δ_C_ 168.1. Detailed long range correlation analysis constructed the *N*-ethyl glycine residue, two anthranilic acid residues and the phenyllactic acid residue into a cyclopeptide with three amide linkage and one ester linkage (Figure 1). The oxygenated methine proton showed a cross peaks to the carbonyl of one of the anthranilic acid residues revealed an ester linkage between anthranilic acid moiety and phenyllactic acid moiety. HMBC correlation between *N-*ethyl protons to the carbonyl of phenyllactic acid residue positioned an amide linkage between *N*-ethyl glycine unit and phenyllactic acid unit. The amide proton showed a HMBC cross peak to the carbonyl of glycine unit, indicating an amide linkage between these two units. The substructure of anthranilic acid dimer was deduced from the observation of HMBC correlation from the amide proton of the unit anthranilic acid I to the carbonyl of unit anthranilic acid II. In order to consolidate the elucidation of the sequencing of four parts, MS/MS experiment was measured. According to the MS and MS/MS spectra (Supplementary Figure S25), the structure of **1** is proposed as a cyclopeptide. In the proposed structure (Figure 2), the amide and ester bonds are the most potential cleavage sites during CID. Based on the MS/MS spectrum, the cleavage pathways are labeled in Figure 2 as well. In details, the cleavages of the C1-N2 amide bond and the C8-N9 amide bond give rises of the fragment of *m/z* 268. The cleavages of the C4-N5 amide bond and the C12-O13 ester bond form the fragment at *m/z* 234. In addition, this fragment can further lose the C4 ketone to produce the fragment of *m/z* 206. Furthermore, two possible pathways will bring the fragment peak of *m/z* 120, including (i) the cleavages of the C4-N5 amide bond and the C8-N9 amide bond and (ii) the cleavages of the C8-N9 amide bond and the C12-O13 ester bond. These two fragmentation pathways produce exactly the same fragment, which could explain that the *m/z* 120 fragment peak is observed as the dominant peak in the MS/MS spectrum (Supplementary Figure S25). Thus, the planar structure of the new cyclopeptide was elucidated as drawn (Chart 1). The assignment of NMR signals of **1** is listed in Table 1.

**Table 1:** NMR Data (500 MHz) for Compound **1** and **2** in CDCl_3_.

Position	1		2	
	δ_C_*^a,b^*, Mult.	δ_H_*^c^*, Mult. (*J* in Hz)	δ_C_*^a,b^*, Mult.	δ_H_*^c^*, Mult. (*J* in Hz)
d-phenyllactic acid				
1	169.6, C		169.8, C	
2	71.7, CH	5.49, dd (8.3, 6.3)	71.5, CH	5.51 t (7.5)
3	37.6, CH_2_	3.46, dd (13.7, 8.3); 3.25, overlap	37.2, CH_2_	3.39, dd (13.6, 7.5); 3.25, (13.6, 7.5)
4	135.9, C		135.5, C	
5/9	129.7, CH	7.34, overlap	129.6, CH	7.31, overlap
6/8	128.7, CH	7.37, overlap	128.7, CH	7.34, overlap
7	127.3, CH	7.34, overlap	127.4, CH	7.31, overlap
anthranilic acid I				
10	167.1, C		167.5, C *^d^*	
11	127.8, C		127.4	
12	129.7, CH	7.63, d (7.6)	129.6, CH	7.62, d (7.8)
13	126.6, CH	7.30, overlap	126.5, CH	7.30, overlap
14	132.5, CH	7.50, t (7.6)	132.7, CH	7.44, t (7.8)
15	125.8, CH	7.22, d (7.6)	126.1, CH	7.19, d (7.8)
16	134.4, C		134.8, C	
NH		8.07, s		8.58, s
anthranilic acid II				
17	167.3, C		167.4, C *^d^*	
18	123.0, C		123.1, C	
19	126.2, CH	7.58, d (7.6)	126.5, CH	7.56, d (7.8)
20	123.1, CH	7.10, t (7.6)	123.2, CH	7.03, t (7.8)
21	132.3, CH	7.46, t (7.6)	132.2, CH	7.40, t (7.8)
22	122.0, CH	8.57, d (7.6)	121.9, CH	8.48, d (7.8)
23	138.2, C		137.9, C	
NH		10.23, s		10.17, s
glycine				
24	168.1, C		167.5, C *^d^*	
25	51.7, CH_2_	5.24, d (15.2); 3.29, d (15.2)	54.1, CH_2_	5.25, d (14.8); 3.10 d (14.8)
26	43.3, CH_2_	3.95, m; 3.27, m	35.9, CH_3_	3.01, s
CH_3_	13.7, CH_3_	1.02, t, (7.2)		

*^a^*, Recorded at 125 MHz; *^b^*, Multiplicities inferred from DEPT and HSQC experiments; *^c^*, Recorded at 500 MHz; *^d^*, Interchangeable.

Compound **2**, [α]^24^_D_ +65 (*c* 0.37, MeOH); UV (MeOH) λ_max_ (log є) 290 (3.32) nm, was obtained as a yellow amorphous solid. The HR-TOF-MS exhibited an ion peak at *m/z* 458.1729 [M + H]^+^ (calcd. for C_26_H_24_N_3_O_5_, 458.1716), indicating that the molecular formula was C_26_H_24_N_3_O_5_ with 17 degrees of unsaturation. The IR and NMR data (Table 1) of **2** were similar to those of **1**, except that *N*-Me glycine unit in **2** replaced the *N-*ethyl glycine in **1**. Detailed 1D and 2D NMR analysis proved that the amino sequence in **2** was the same as that in **1**. The absolute configuration of the phenyllactic acid moiety in clavatustides A (**1**) and B (**2**) were determined by analysis of the CD spectrum of **1** and **2**. The low-wavelength Cotton effect of the chromophore around 221 of both compounds were negative (Supplementary Figures S23 and S24), revealing a 2-*R* configuration in **1** and **2** [13]. This inference was also confirmed by the measurement of their positive optical rotation and the analyses of acidic hydrolysates through a chiral HPLC column, when compared with the authentic d-(+)-phenyllactic acid. Thus, the structures of compounds **1** and **2** were determined as two novel cyclopeptides, and named as clavatustides A (**1**) and B (**2**).

**Figure 3:**
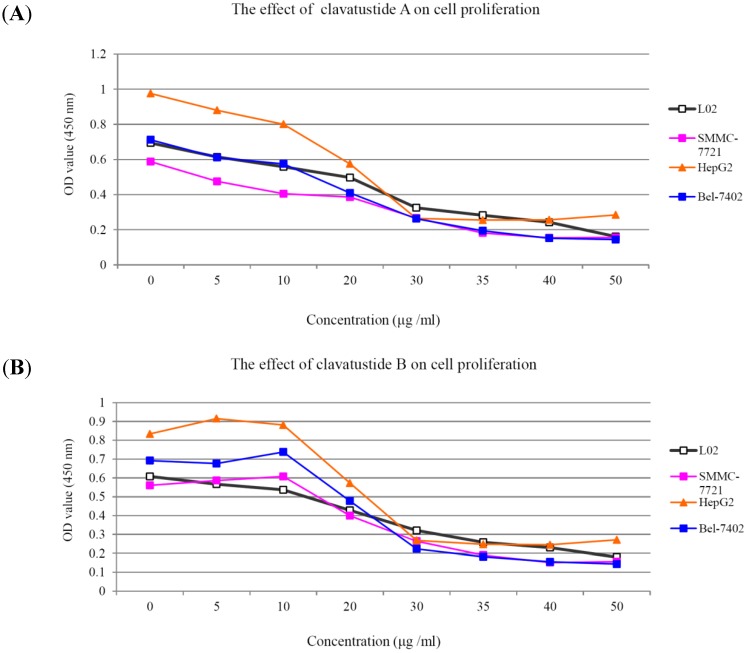
The suppression of cell proliferation of clavatustide A (**A**) and clavatustide B (**B**) in a dose-dependent manner.

**Figure 4:**
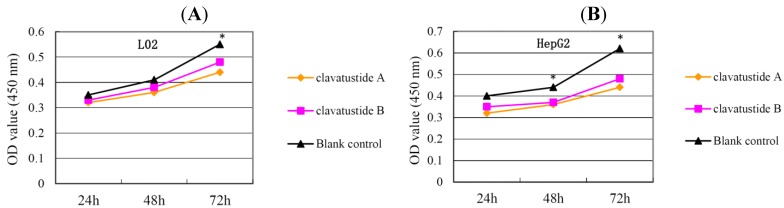
The suppression of cell proliferation of clavatustide A and clavatustide B in L02 (**A**) and HepG2 (**B**) was in a time dependent manner. * *P* < 0.05 if treatment group *vs.* control group. L02, liver cells; HepG2, SMMC-7721 and Bel-7402, human hepatocellular carcinoma (HCC) cell lines.

After several purification steps, the ^1^H NMR spectra of clavatustides A and B still contained some unknown impurities. Nevertheless, the HPLC analysis of the fractions obtained showed a purity of >95%, which were submitted to the biological tests. To assess the potential treatment value of clavatustides A and B in liver cancer, the effect of clavatustide A/clavatustide B on cell proliferation in hepatocellular carcinoma (HCC) cell lines (HepG2, SMMC-7721 and Bel-7402) and normal human hepatocytes (L02 cells) were investigated. When cells were treated with different concentration for 48 h, the proliferations of all cell lines were suppressed in a dose dependent manner (Figure 3). Compared with normal human hepatocytes (L02 cells), all HCC cell lines (HepG2, SMMC-7721 and Bel-7402) showed a lower Max effect dose (30 μg/mL *vs.* 50μg/mL, *P* < 0.05) and IC_50_ dose (15 μg/mL *vs.* 25 μg/mL, *P* < 0.05). HepG2 was the most sensitive cell lines to the compounds and was used for the following experiments. When HepG2 and L02 cells were treated with 15 μg/mL of compounds for different duration, the proliferations of cells were suppressed in a time dependent manner. In HepG2 cells, the cell numbers became significant different between treatment group and control group at 48 h (Figure 4B, *P* < 0.05). While in L02 cells, the significance appeared at 72 h after treatment (Figure 4A, *P* < 0.05). It indicated that HCC cell lines were more sensitive than normal human hepatocytes in the drug-induced cell proliferation suppression. The results showed that clavatustide A and B sharply and significantly reduced the HCC cell proliferation in a time-dependent manner.

**Figure 5:**
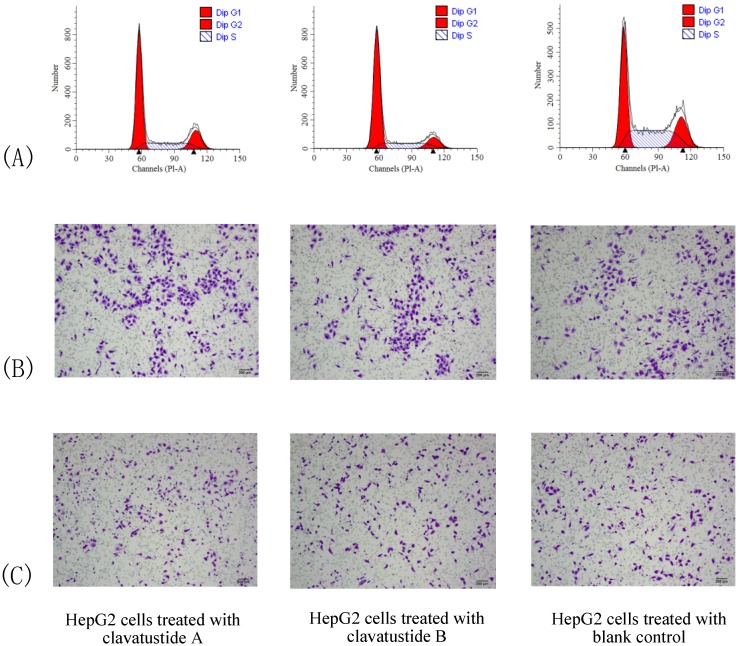
The comparison of cell cycle (**A**), cell migration (**B**) and invasion (**C**) between HepG2 cells treated with clavatustide A/clavatustide B and the blank control. **A**: Clavatustide A/clavatustide B treated cells showed significantly accumulation in G1 phase and reduction of cells in S phase, compared with control group (*P* < 0.05). **B** and **C**: The counted migrated/invaded cell numbers did not differ significantly between treated groups and control group.

To further explore the mechanism of proliferation suppress effect, cell cycle analysis was performed. HepG2 cells were treated with clavatustide A/clavatustide B with the purity >95% (determined by HPLC) at 15 μg/mL for 48 h (Table 2). An accumulation of HepG2 cells in G1 phase and reduction of cells in S phase were found (*P* < 0.05, Figure 5A). Clavatustide A and B induced a G1 arrest and suppression of G1/S phase transition, and subsequently inhibited proliferation in human HCC cells. Although the detailed mechanism still need to be explored and is in progress, the important finding provides a promising perspective of marine cyclodepsipeptides containing an anthranilic acid dimer and a d-phenyllactic acid residues on anti-cancer therapy.

**Table 2:** G1 arrest and S phase reduction of clavatustides A and B on HepG2 cells.

	G1 Phase	S Phase	G2 Phase
Clavatustide A	59.17%	17.73%	23.10%
Clavatustide B	67.57%	11.92%	20.52%
Blank control	41.03%	19.49%	39.48%

Cell invasion is a crucial step in metastasis. Therefore, the effect of clavatustide A/clavatustide B on cell migration and invasion was evaluated. Compared with the blank control, cells treated with clavatustide A/clavatustide B did not show significant change in migration/invasion abilities (Figure 5B,C).

## 3. Experimental Section

### 3.1. General Experimental Procedures

Optical rotations were recorded on a Perkin-Elmer-341 polarimeter. The IR spectra (CHCl_3_) were run on a NicoletAvatar-360FT-IR spectrometer. ^1^H NMR (500 MHz) and ^13^C NMR (125 MHz) spectra were measured at 25 °C on a Bruker AVANCE DMX 500 NMR spectrometer with TMS as internal standard. TOF-MS were recorded on an Agilent 6210 TOF LC/MS spectrometer. ESIMS were recorded on an Agilent 6460 Triple Quad LC/MS. TLC was performed using Merck precoated plates (Silica gel 60 F254, Merck, Darmstadt, Germany) of 0.25 mm thickness. Prep. HPLC was performed on an Agilent-1100 system equipped with a Venusil MP-C_18_ column (10 mm × 250 mm, Agela Technologies, Tianjin, China). Sephadex LH-20 (Amersham Biosciences, Piscataway, NJ, USA) was used for column chromatography.

### 3.2. Fungal Cultivation

The fungus *Aspergillus clavatus* C2WU was separated isolated from hydrothermal vent crab *Xenograpsus testudinatus*, which was collected from Kueishantao, Taiwan, and identified by its ITS-5.8s rDNA sequences. The surface of the shell of the crab sample was rinsed three times with sterile artificial seawater. The surface of the shell tissue was excised with a sterile scalpel and scraped into powder. Powder was homogenized using a blender containing 20 mL sterile natural seawater in aseptic conditions. The resulting homogenate was diluted with sterile seawater (1:5, 1:25, 1:125, 1:625). Under sterile conditions, 200 µL of each dilution was inoculated in quadruplicate onto GYP, containing 1.0 g glucose, 0.1 g yeast extract, 0.5 g peptone, 15 g agar per liter seawater. The plates were incubated at room temperature for 1–3 weeks until the morphology of fungi could be distinguished. Each isolate was picked. Pure strains of *A. clavatus* C2WU were isolated by reinoculation on agar plates. Subcultures of the organism are deposited at China Center for Type Culture Collection (CCTCC) (No. CCTCC M 201342). The fungus was grown in the culture medium consisting of (g L^−1^) sucrose (20.0 g), yeast extract (10.0 g), malt extract (20 g), MgSO_4_·7H_2_O (0.4 g), and KH_2_PO_4_ (0.4 g) in sterilized and filtrated seawater. The fermentations were carried out at 24 °C for 50 days.

### 3.3. Extraction and Isolation

The whole culture of the broth of *A. clavatus* C2WU (30 L) were filtered. The air-dried mycelia of control (253 g) were extracted at room temperature with MeOH (3 × 1 L), respectively. The extracts were evaporated in *vacuo* to afford a gummy residue (13 g). The residues were partitioned in H_2_O (500 mL) and extracted with EtOAc (3 × 3 L). The broth (30 L) was extracted with EtOAc (3 × 20 L). Both EtOAc extracts from mycelia and broth were combined (11 g). The concentrated EtOAc extract was adsorbed onto Si gel (20 g) and subjected to chromatography on Si gel (4 × 50 cm, 800 g, 200–300 mesh), eluting with petroleum ether/EtOAc gradient mixtures (flow rate 3 mL/min). Five main fractions were obtained by checking with TLC and combined (2 L/fraction). The fourth fraction was subjected to preparative HPLC (flow rate 8 mL/min, UV detector 254 nm), using MeOH:H_2_O (15:85) as eluent, to afford fraction A and B. Fraction A and B were applied to a Sephadex LH-20 column (1 × 80 cm, 38 g, Amersham Biosciences, Piscataway, NJ, USA, eluted with MeOH), and then preparative HPLC (flow rate 8 mL/min, UV detector 254 nm, MeOH:H_2_O 15:85) to afford two compounds **1** (13.1 mg, *t_R_* 28 min) and **2** (14.1 mg, *t_R_* 17 min). These two compounds were used for the biological tests.

Clavatustide A (**1**): yellow amorphous solid; [α]^24^_D_ +22 (*c* 0.10, MeOH); M.p. 187–188 °C; UV (MeOH) λ_max_ (log є) 287 (3.11) nm; IR ν_max_ 3281, 2928, 2854, 1726, 1656, 1602, 1517, 1300, 1256, 1081, 754, 700 cm^−1^; ^1^H NMR and ^13^C NMR, see Table 1; ESIMS *m/z* 472 [M + H]^+^; HR-TOF-MS *m/*z 472.1871 [M + H]^+^ (calcd. for C_27_H_26_N_3_O_5_, 472.1872).

Clavatustide B (**2**): yellow amorphous solid; [α]^24^_D_ +65 (*c* 0.37, MeOH); M.p. 193–194 °C; UV (MeOH) λ_max_ (log є) 290 (3.32) nm; IR ν_max_ 3300, 2931, 1717, 1659, 1601, 1515, 1446, 1320, 1077, 753, 701 cm^−1^; ^1^H NMR and ^13^C NMR, see Table 1; ESIMS *m/z* 458 [M + H]^+^; HR-TOF-MS *m/*z 458.1729 [M + H]^+^ (calcd. for C_26_H_24_N_3_O_5_, 458.1716).

### 3.4. Cell Proliferation Assay

Human immortalized liver cells (L02) and human hepatocellular carcinoma (HCC) cell lines (HepG2, SMMC-7721 and Bel-7402) were purchased from the Cell Bank of the Chinese Academy of Sciences (Shanghai, China), and cultured in DMEM with 10% fetal bovine serum at 37 °C in a humidified atmosphere containing 5% CO_2_. Cell viability was determined by cell counting kit-8 (Dojindo, Tokyo, Japan) assay. Briefly, cells were plated in 96-well plate (2 × 103 cells per well) and incubated for 24 h. Then the cells were treated with clavatustide A or clavatustide B for different time points and concentrations. After incubation, CCK-8 solution was added and the absorbance at 450 nm was measured according to the manufacturer's instructions. The experiment was independently repeated three times.

### 3.5. Cell Cycle Assay

Cell cycle analysis was performed using flow cytometry according to the manufacturer’s instructions. Briefly, cells were washed and fixed in 70% cold ethanol overnight at 4 °C. Then cell was mixed with 0.5 mL DNA Prep LPR (Coulter DNA-Prep Reagents kit, Beckman Coulter, Fullerton, CA, USA) in the dark for 20 min. Cell cycle analysis was performed on the same flow cytometer (Cytomics FC 500, Beckman Coulter, Fullerton, CA, USA). ModFit LT software was used to calculate the percentage of cell population in each phase.

### 3.6. Migration and Invasion Assay

Migration and invasion assay was performed with a polycarbonate membrane transwell (Millipore, Billerica, MA, USA) with (invasion assay)/without (migration assay) an insert coated with matrigel (BD Bioscience, San Jose, CA, USA). Cells were treated with clavatustide A or clavatustide B for 48 h. A total of 5 × 104 cells were suspended in 200 μL of serum-free medium and seeded into the upper chamber, while 600 μL of DMEM containing 10% FBS was added to the lower chamber. After incubation for 48 h, cells on the upper surface were removed with cotton swabs, and the membranes were fixed in methanol and stained with 0.5% crystal violet. The cells on the bottom surface were counted in randomly selected fields with microscope (200×). The experiment was independently repeated three times.

## 4. Conclusions

Marine fungi are a promising source for the discovery of novel natural products [2]. In this study, the structural uniqueness and the bioactivities of the two cyclodepsipeptides, which contain an unusual anthranilic acid dimer and a d-phenyllactic acid residues confirmed that natural products from the extreme environment such as hydrothermal vents organism are of considerable chemical diversity and potent biological activities. Since cyclopeptides sclerotides A and B with an anthranilic acid unit were produced in a nutrient-limited hypersaline cultural medium, and clavatustide A and B were produced by a hydrothermal vent fungus, it is tentatively deduced that anthranilic acid monomer or dimer in cyclopeptides might be a stress driven product or has anti-stress functions. Clavatustide A and B are structurally unique; their simplicity would provide opportunities to design and synthesize new analogs that could improve the biological activity of these compounds.

## Supplementary Files

Supplementary File 1 Supplementary Information (PDF, 1569 KB)
