# Supplementary material for: Two Novel Hepatocellular Carcinoma Cycle Inhibitory Cyclodepsipeptides from a Hydrothermal Vent Crab-Associated Fungus Aspergillus clavatus C2WU
Source: Mar Drugs. 2013 Dec 2;11(12):4761–72. doi: 10.3390/md11124761 (PMC3877885; doi:10.3390/md11124761)

Supplementary Information

Figure S1. ^{1}H NMR in $CDCl_3$ for compound **1**.
Figure S2. ^{1}H NMR-2 in $CDCl_3$ for compound **1**.
Figure S3. ^{13}C NMR in $CDCl_3$ for compound **1**.
Figure S4. DEPT in $CDCl_3$for compound **1**.
Figure S5. COSY in $CDCl_3$ for compound **1**.
Figure S6. COSY-2 in $CDCl_3$ for compound **1**.
Figure S7. HSQC in $CDCl_3$ for compound **1**.
Figure S8. HMBC in $CDCl_3$ for compound **1**.
Figure S9. HMBC-2 in $CDCl_3$ for compound **1**.
Figure S10. ^{1}H NMR in $CDCl_3$ for compound **2**.
Figure S11. ^{1}H NMR-2 in $CDCl_3$ for compound **2**.
Figure S12. ^{13}C NMR in $CDCl_3$ for compound **2**.
Figure S13. COSY in $CDCl_3$ for compound **2**.
Figure S14. COSY-2 in $CDCl_3$ for compound **2**.
Figure S15. DEPT in $CDCl_3$ for compound **2**.
Figure S16. HSQC in $CDCl_3$ for compound **2**.
Figure S17. HMBC in $CDCl_3$ for compound **2**.
Figure S18. HMBC-2 in $CDCl_3$ for compound **2**.
Figure S19. IR for compound **1**.
Figure S20. IR for compound **2**.
Figure S21. HR-TOF-MS for compound **1**.
Figure S22. HR-TOF-MS for compound **2**.
Figure S23. CD for compound **1**.
Figure S24. CD for compound **2**.
Figure S25. CID for compound **1**.

Figure S1. ¹H NMR in $CDCl_3$ for compound **1**.

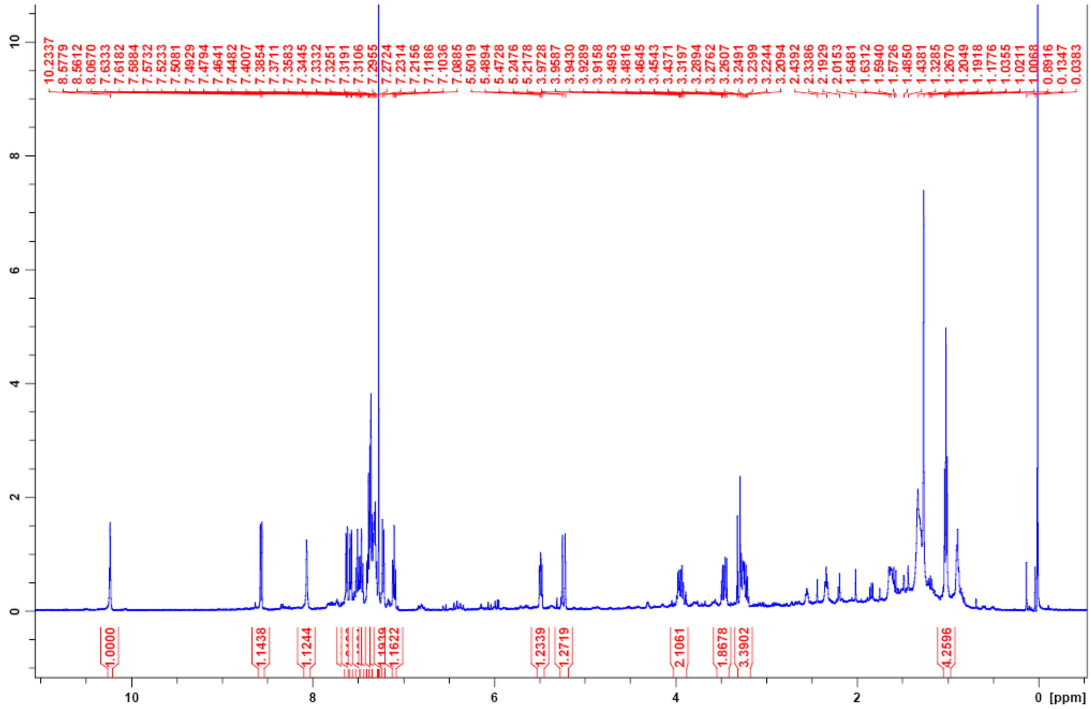

Figure S2. ¹H NMR-2 in $CDCl_3$ for compound **1**.

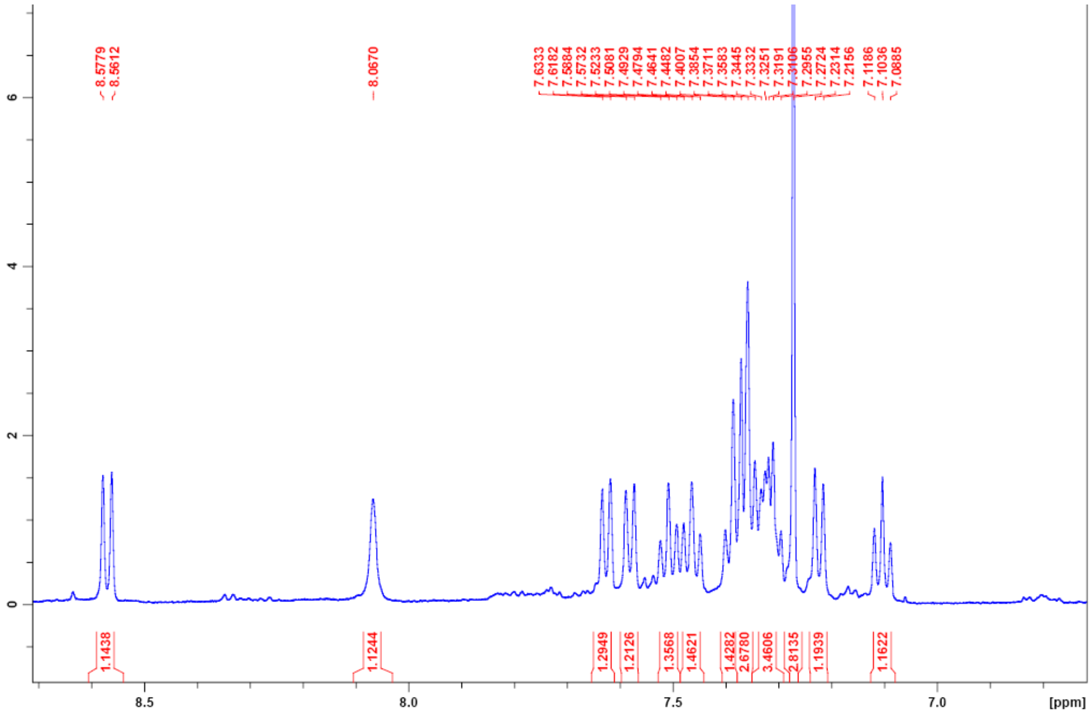

Figure S3. ^{13}C NMR in $CDCl_3$ for compound **1**.

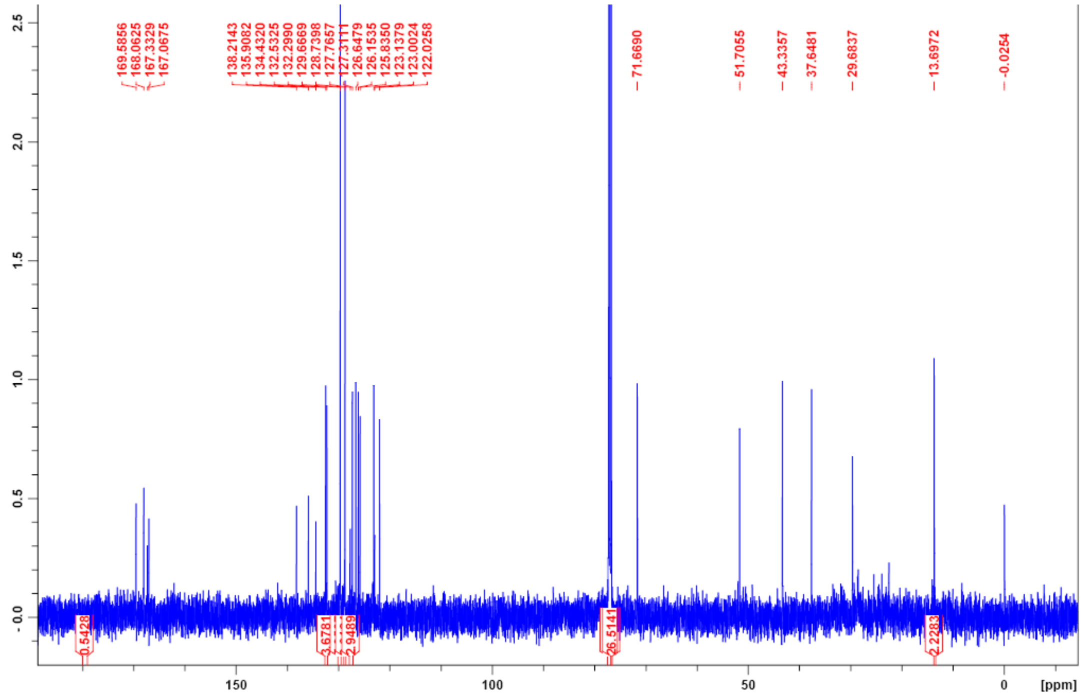

Figure S4. DEPT in $CDCl_3$for compound **1**.

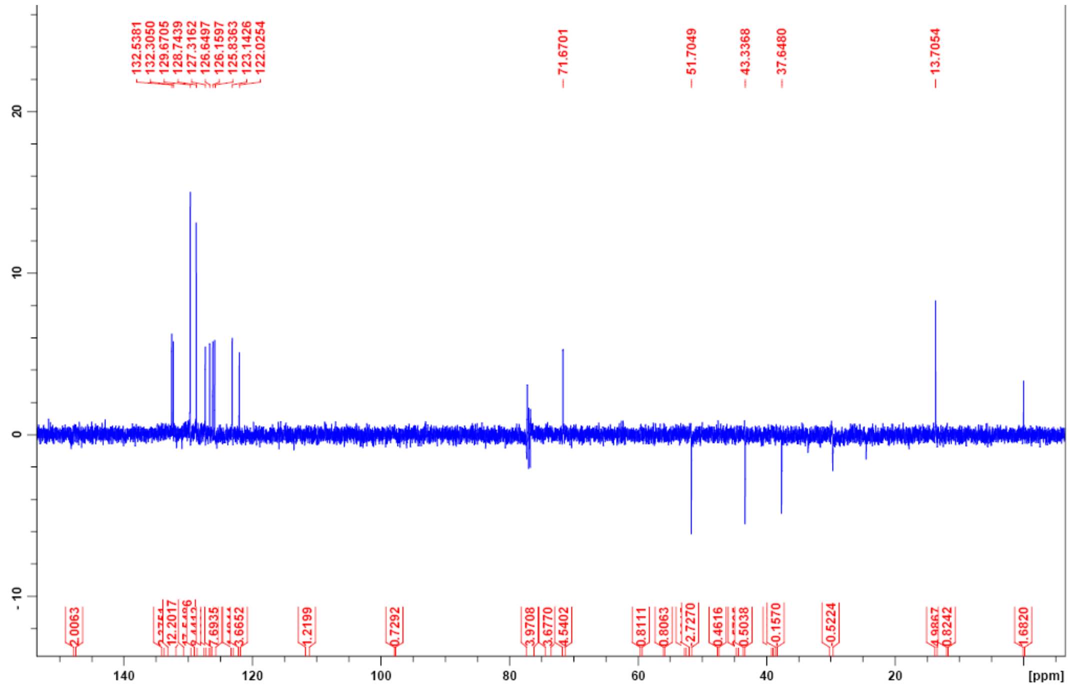

Figure S5. COSY in $CDCl_3$ for compound **1**.

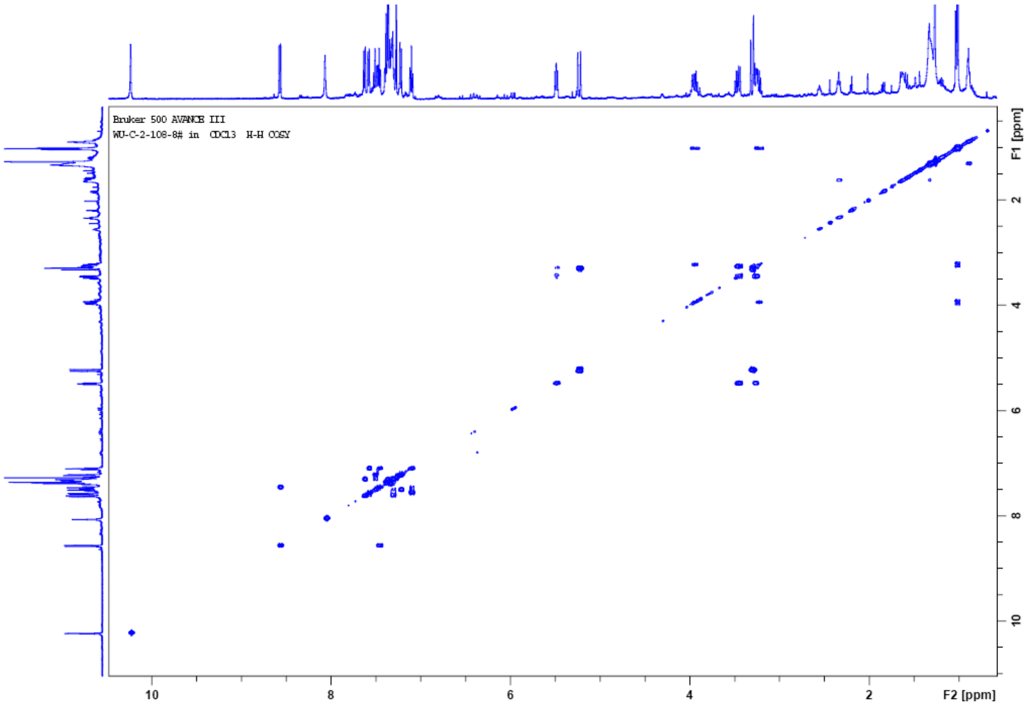

Figure S6. COSY-2 in $CDCl_3$ for compound **1**.

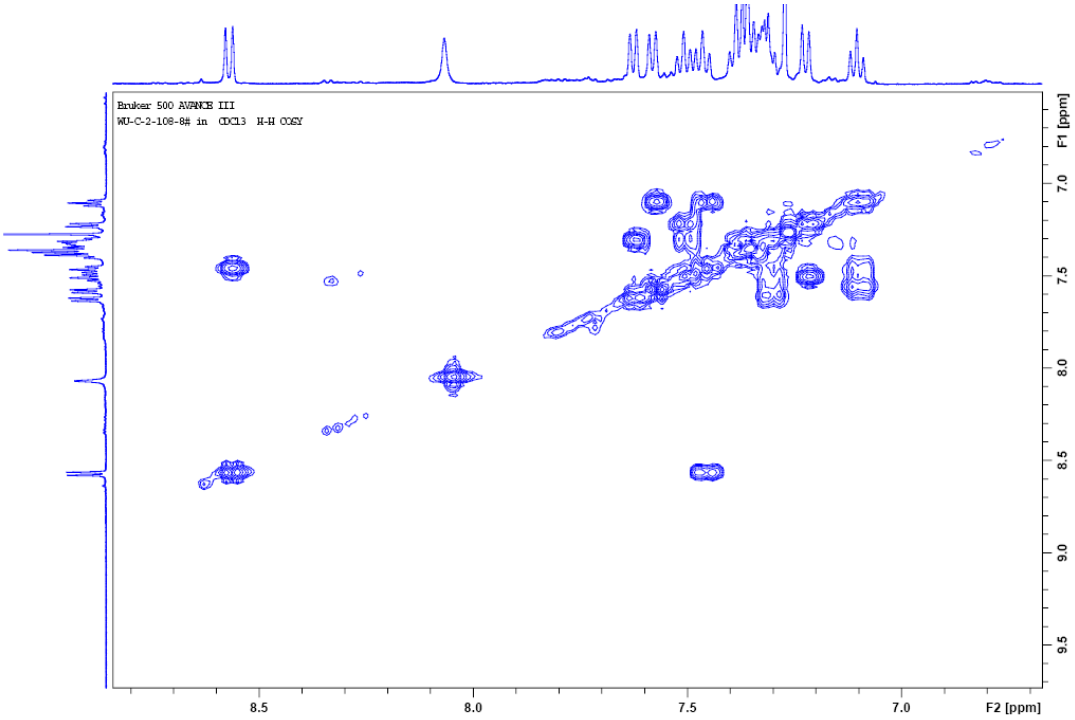

Figure S7. HSQC in $CDCl_3$ for compound **1**.

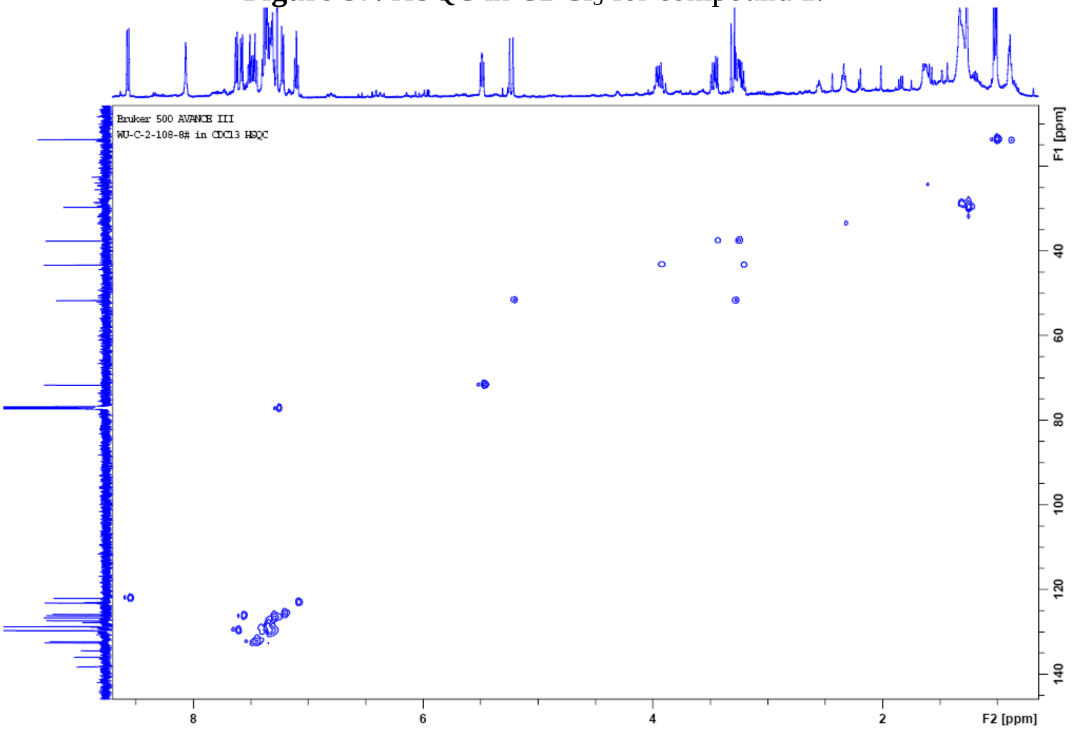

Figure S8. HMBC in $CDCl_3$ for compound **1**.

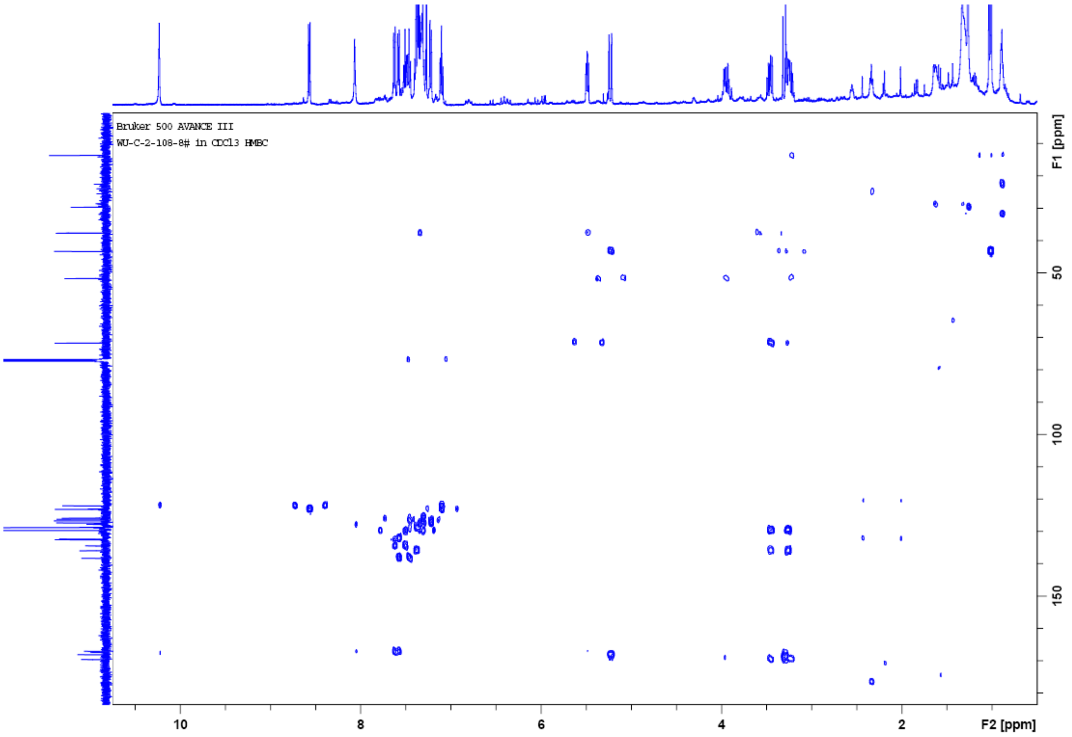

Figure S9. HMBC-2 in $CDCl_3$ for compound **1**.

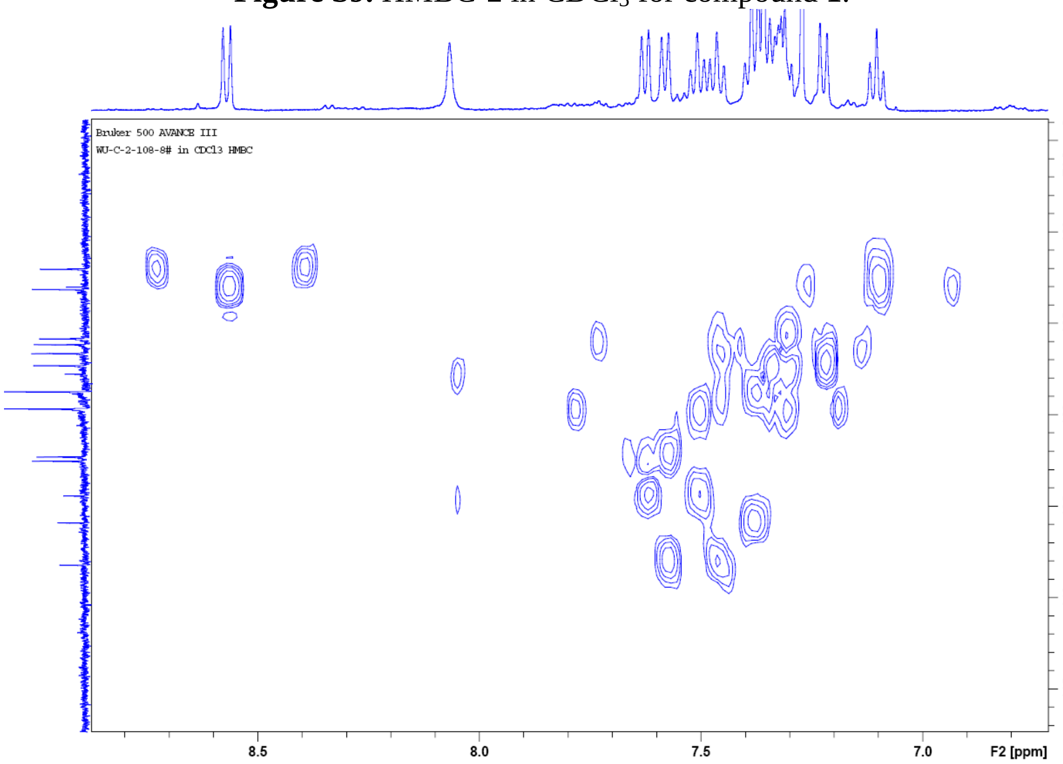

Figure S10. ^{1}H NMR in $CDCl_3$ for compound **2**.

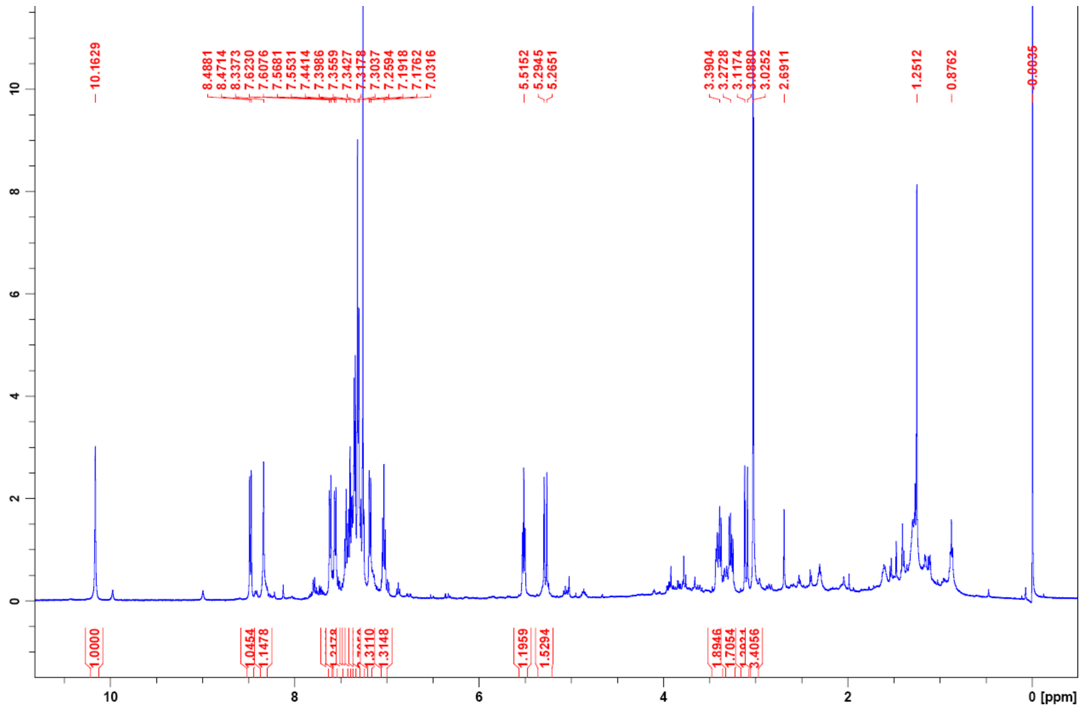

Figure S11. ^{1}H NMR-2 in $CDCl_3$ for compound **2**.

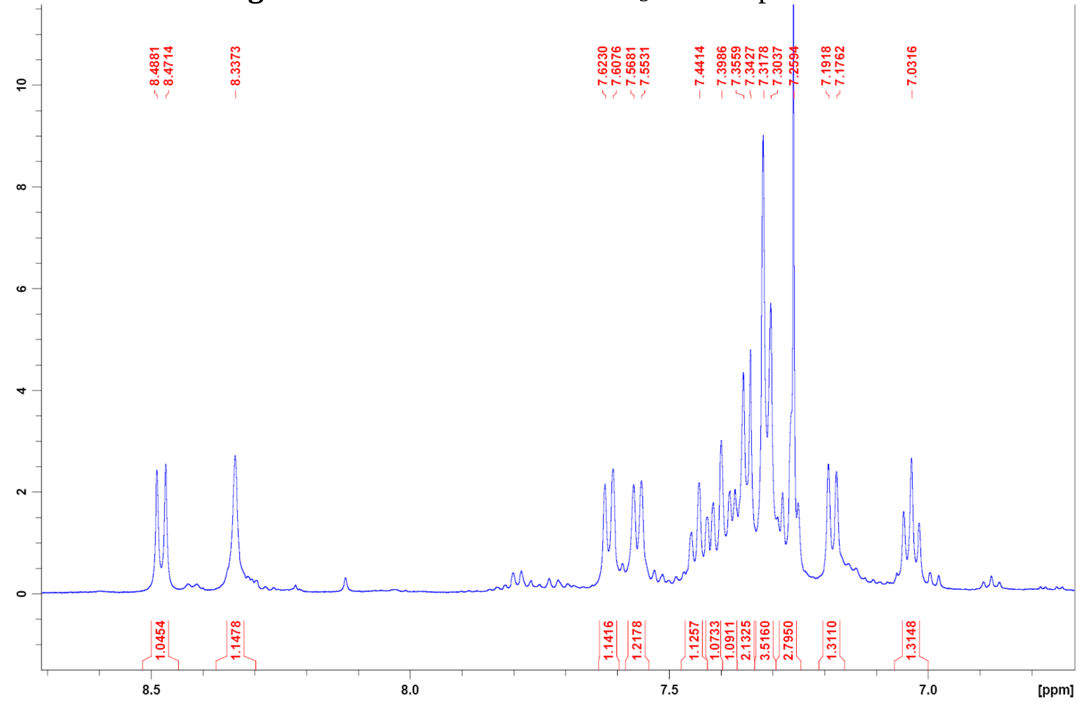

Figure S12. ^{13}C NMR in $CDCl_3$ for compound **2**.

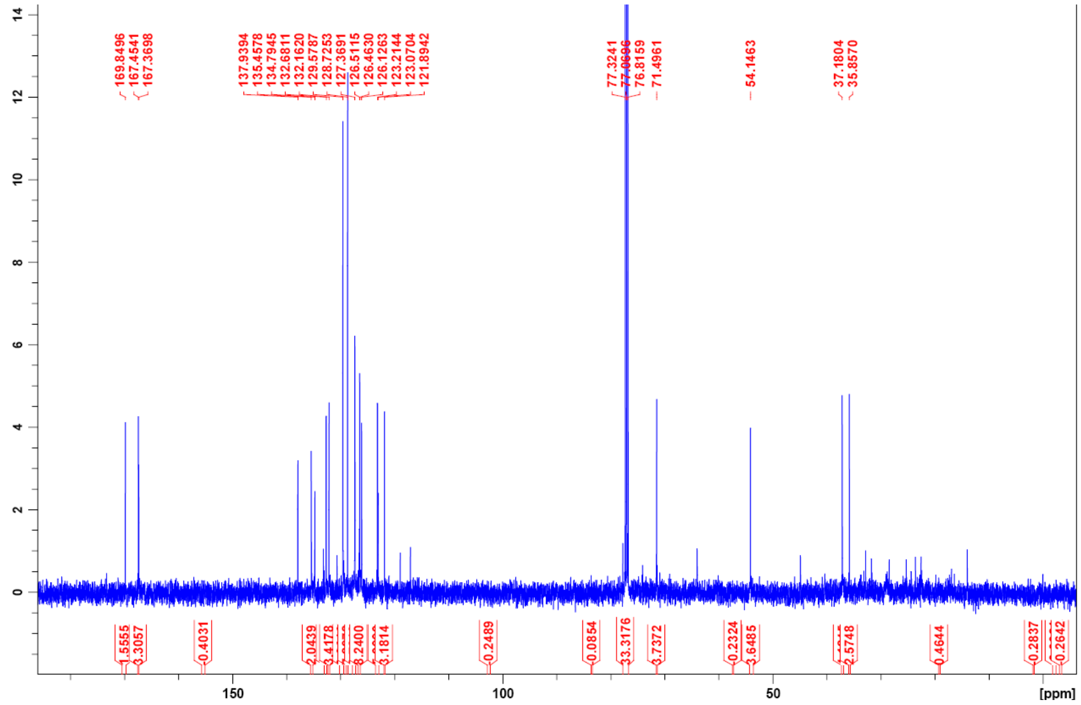

Figure S13. COSY in $CDCl_3$ for compound **2**.

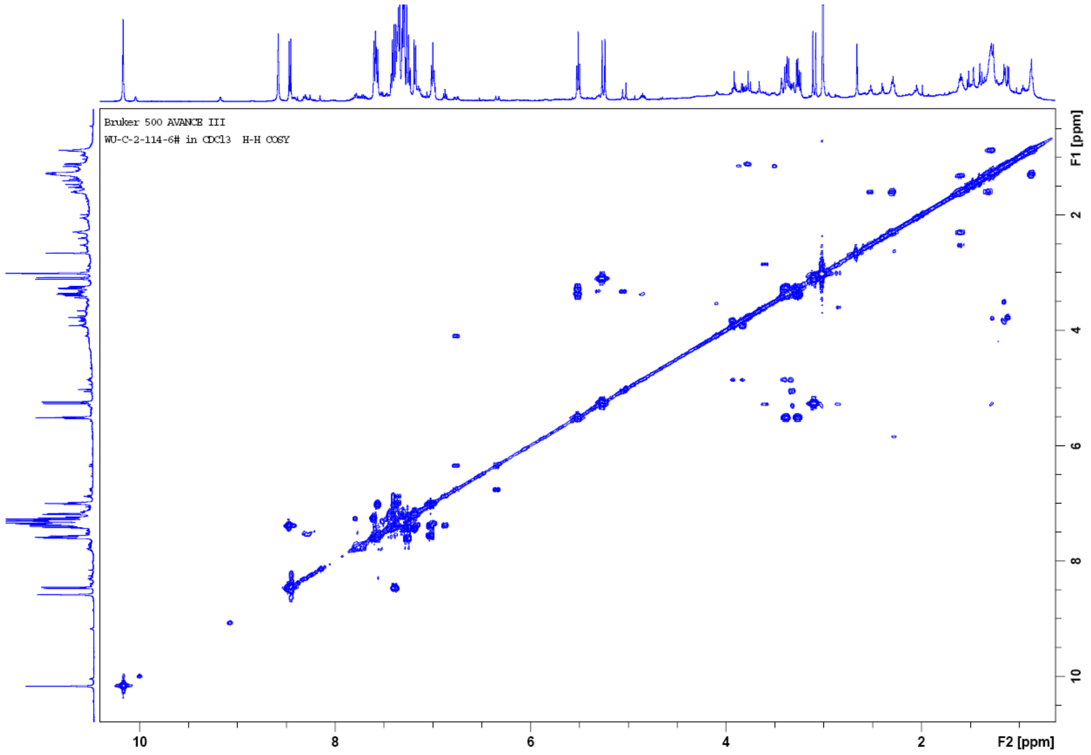

Figure S14. COSY-2 in $CDCl_3$ for compound **2**.

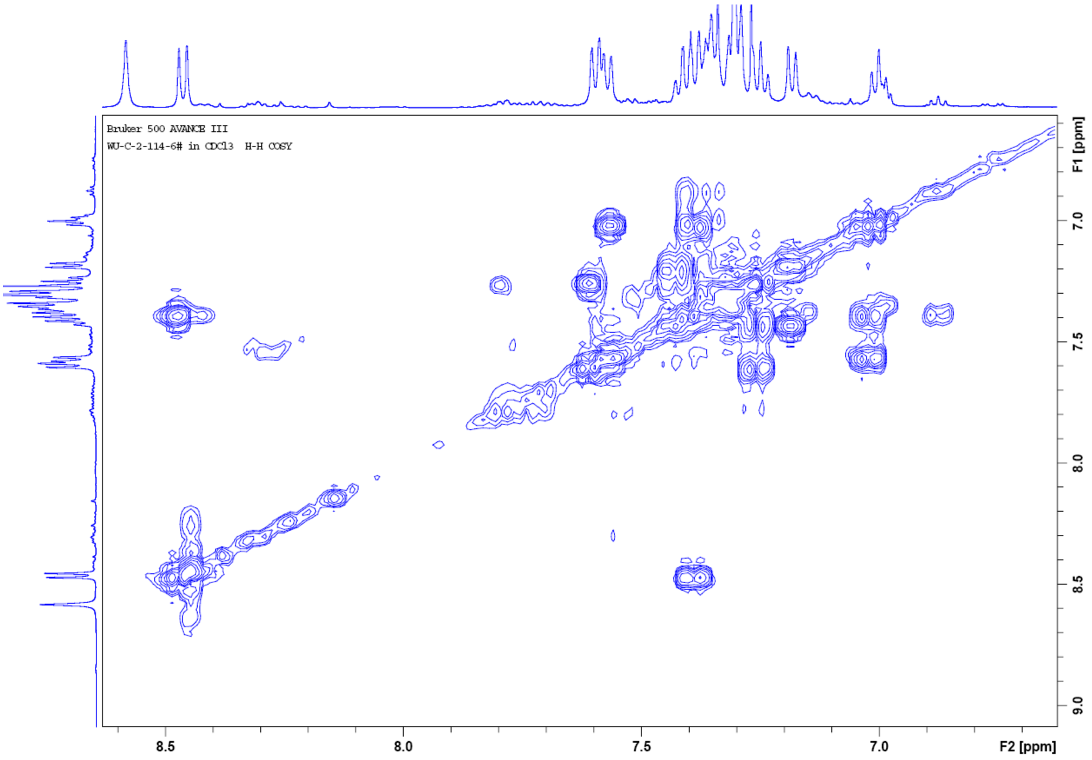

Figure S15. DEPT in $CDCl_3$ for compound **2**.

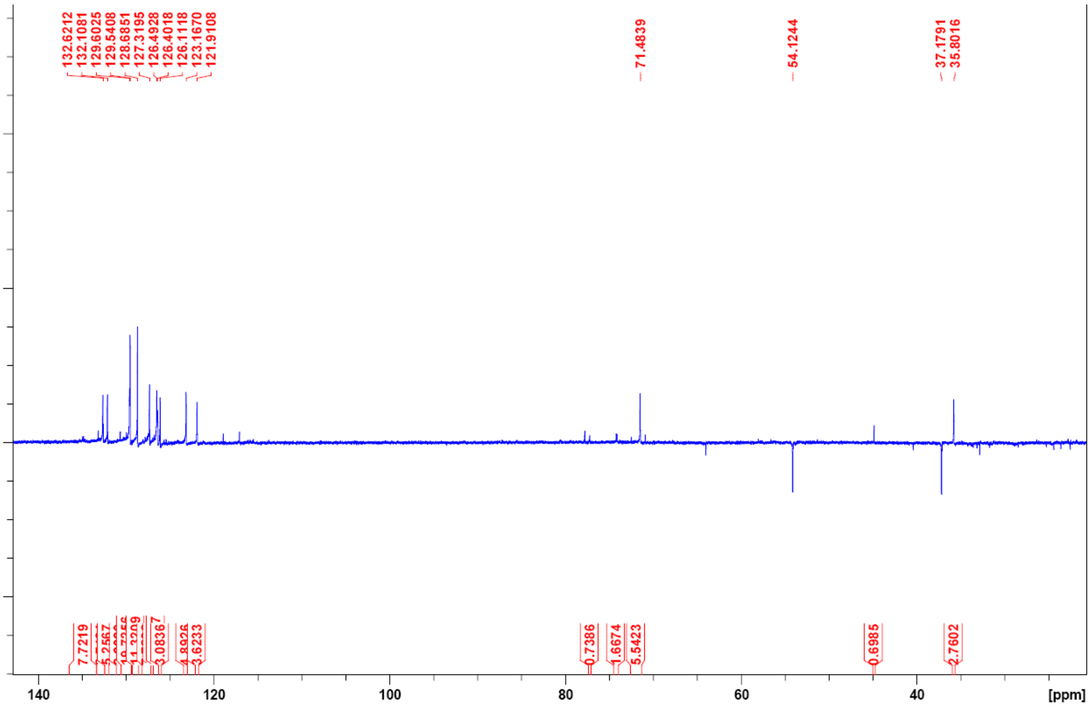

Figure S16. HSQC in $CDCl_3$ for compound **2**.

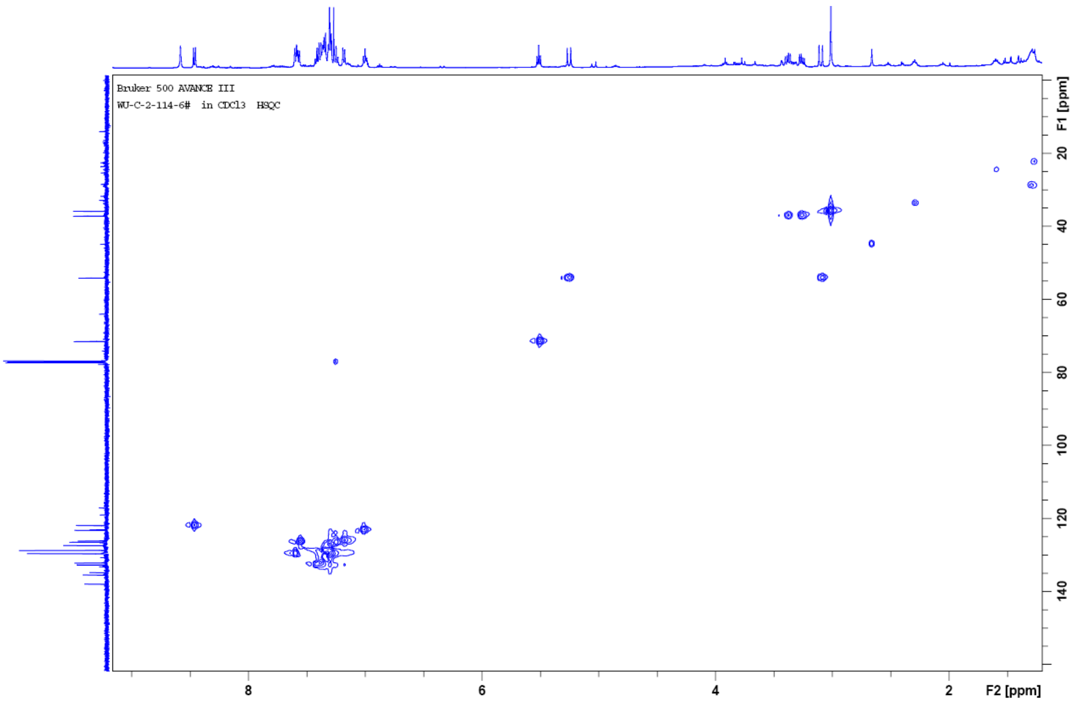

Figure S17. HMBC in $CDCl_3$ for compound **2**.

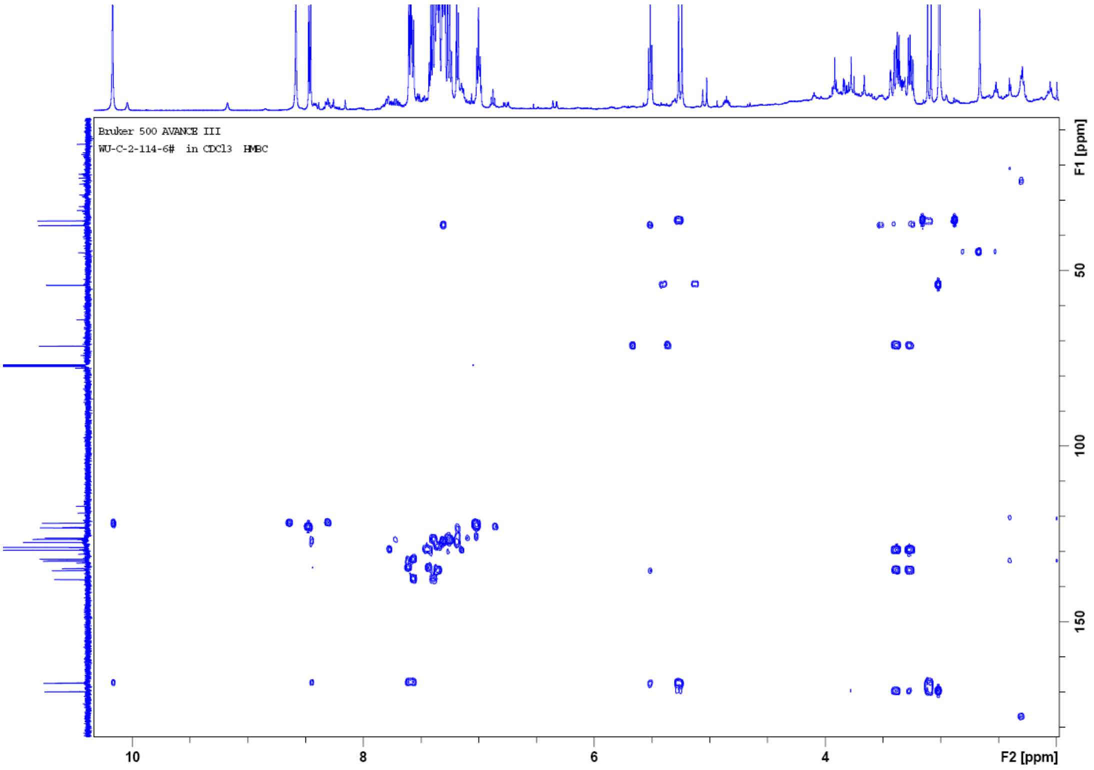

Figure S18. HMBC-2 in $CDCl_3$ for compound **2**.

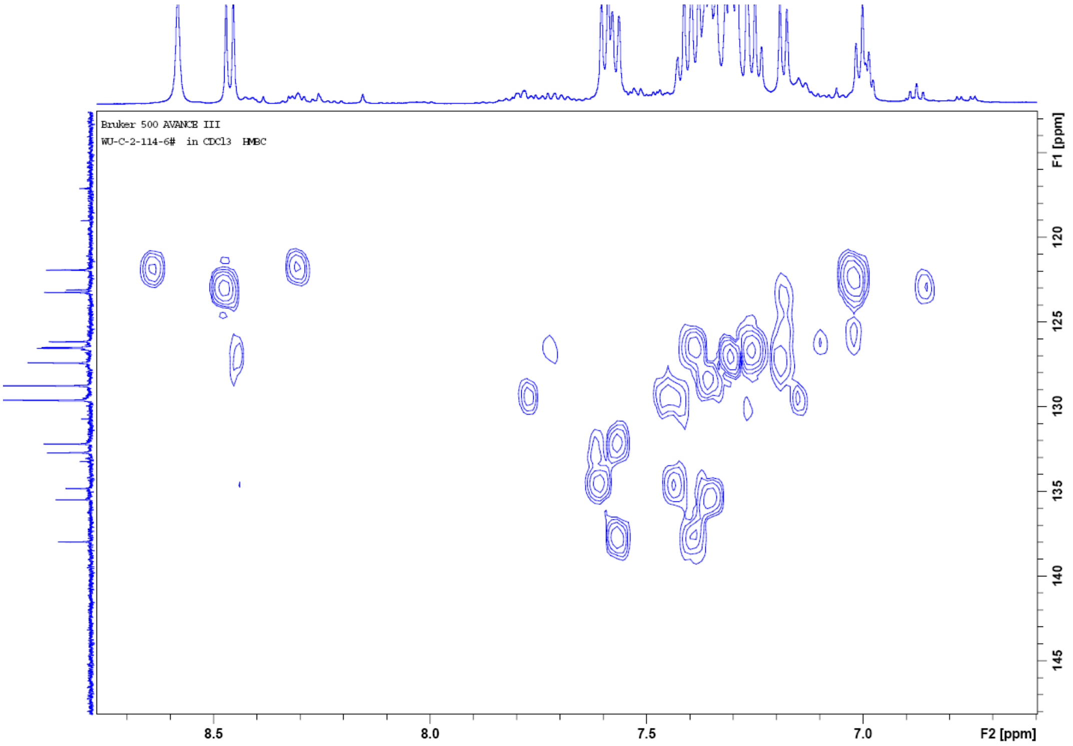

Figure S19. IR for compound **1**.

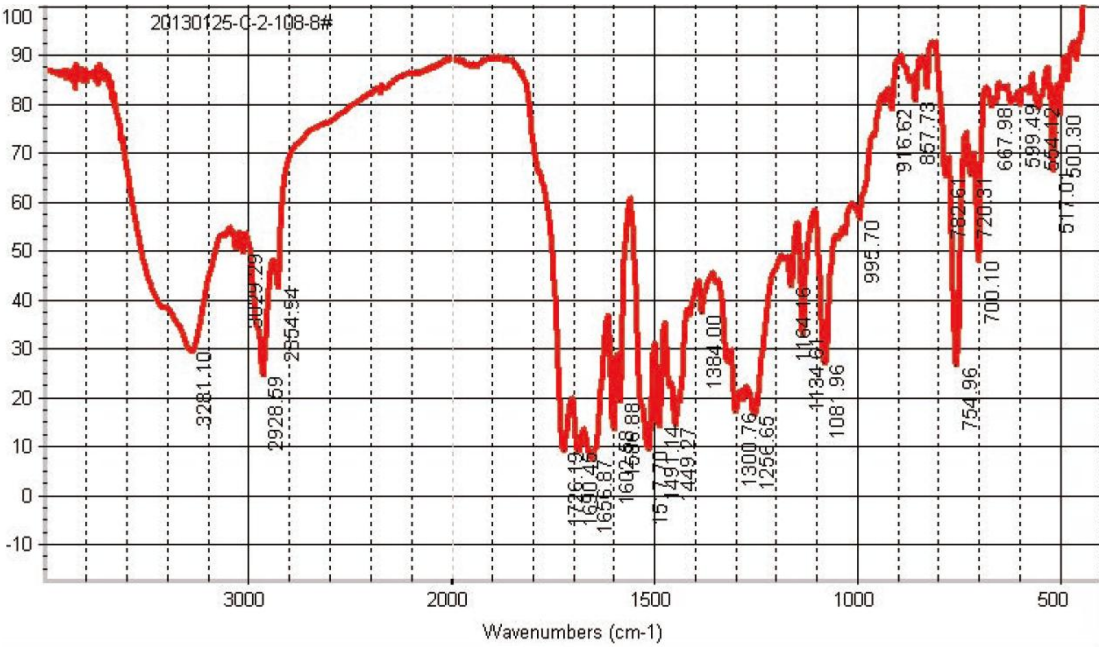

Figure S20. IR for compound **2**.

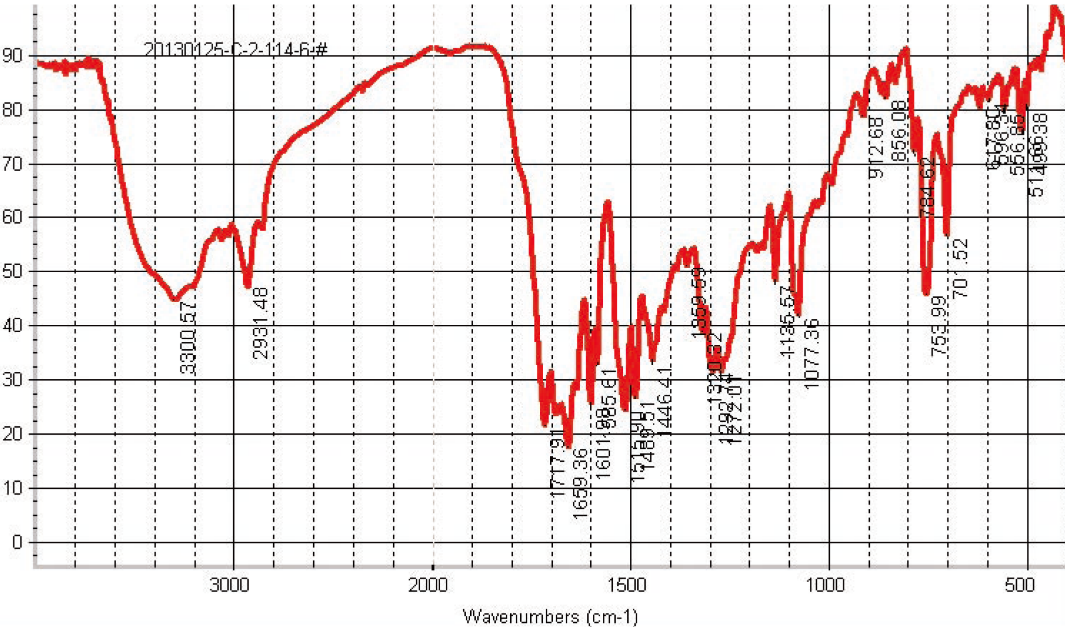

Figure S21. HR-TOF-MS for compound **1**.

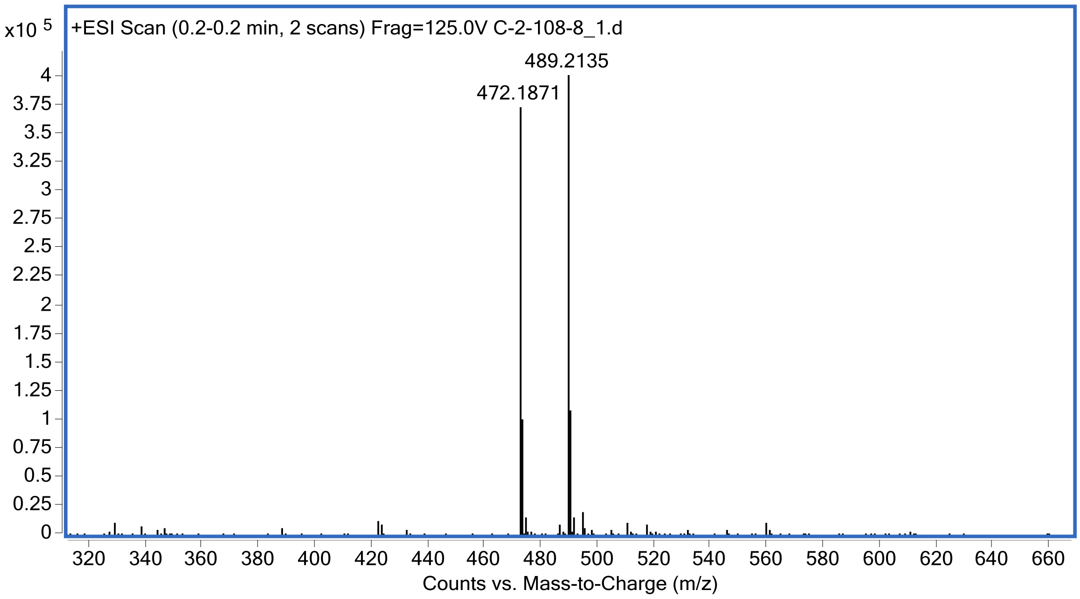

Figure S22. HR-TOF-MS for compound **2**.

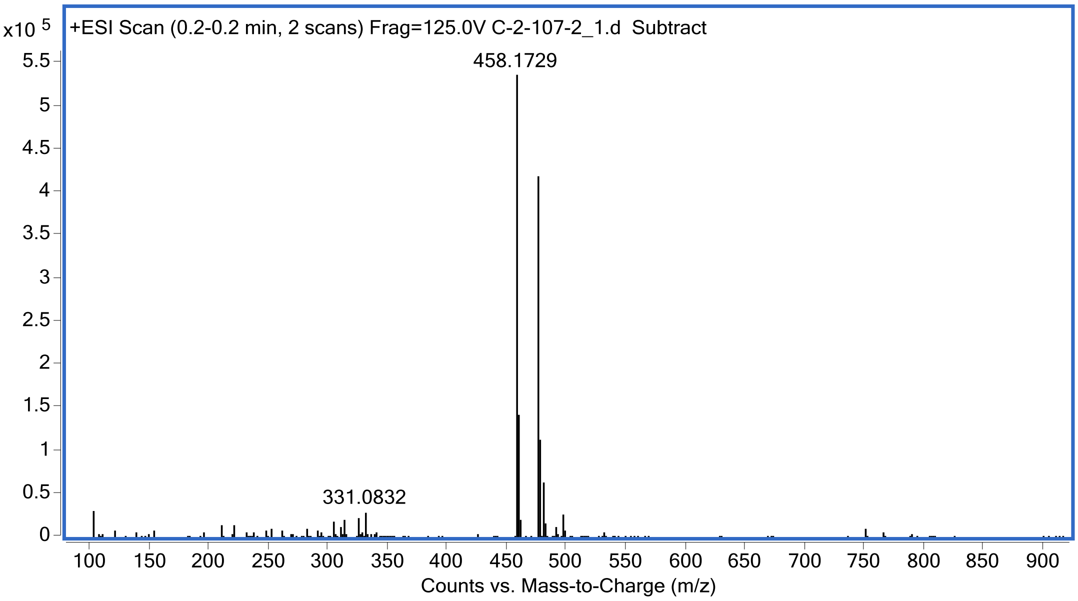

Figure S23. CD for compound **1**.

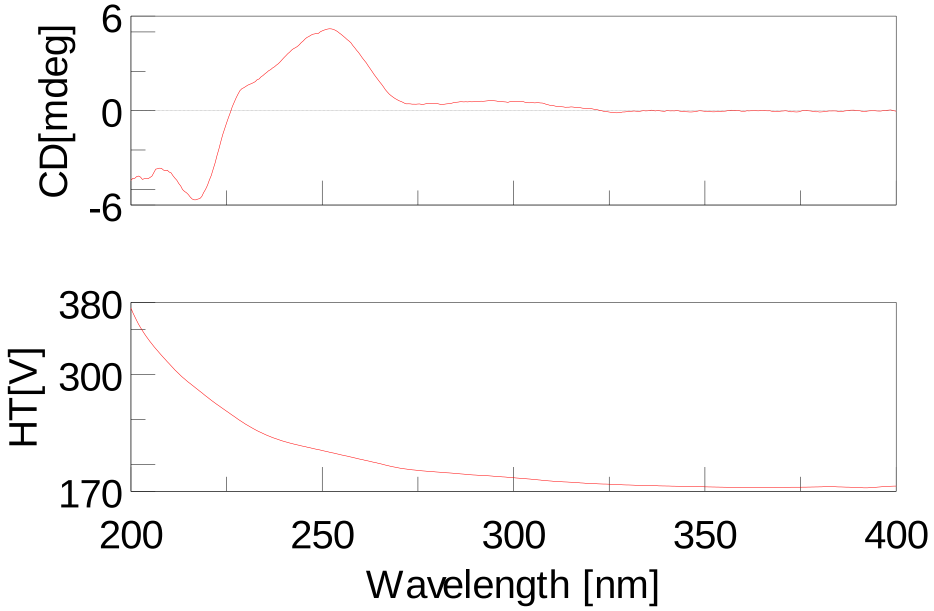

Figure S24. CD for compound **2**.

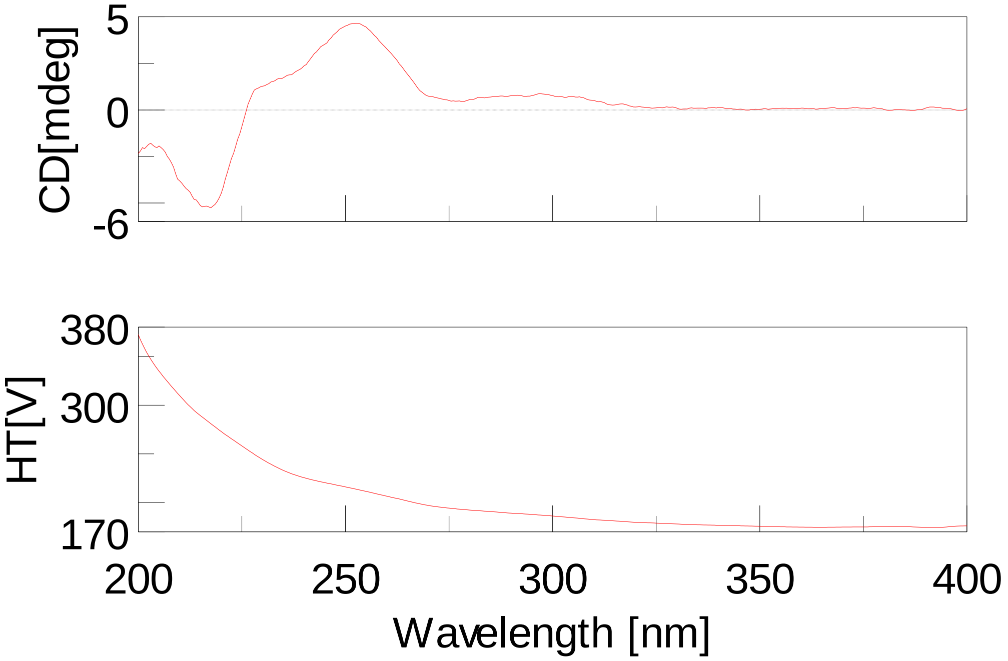

Figure S25. CID for compound **1**.

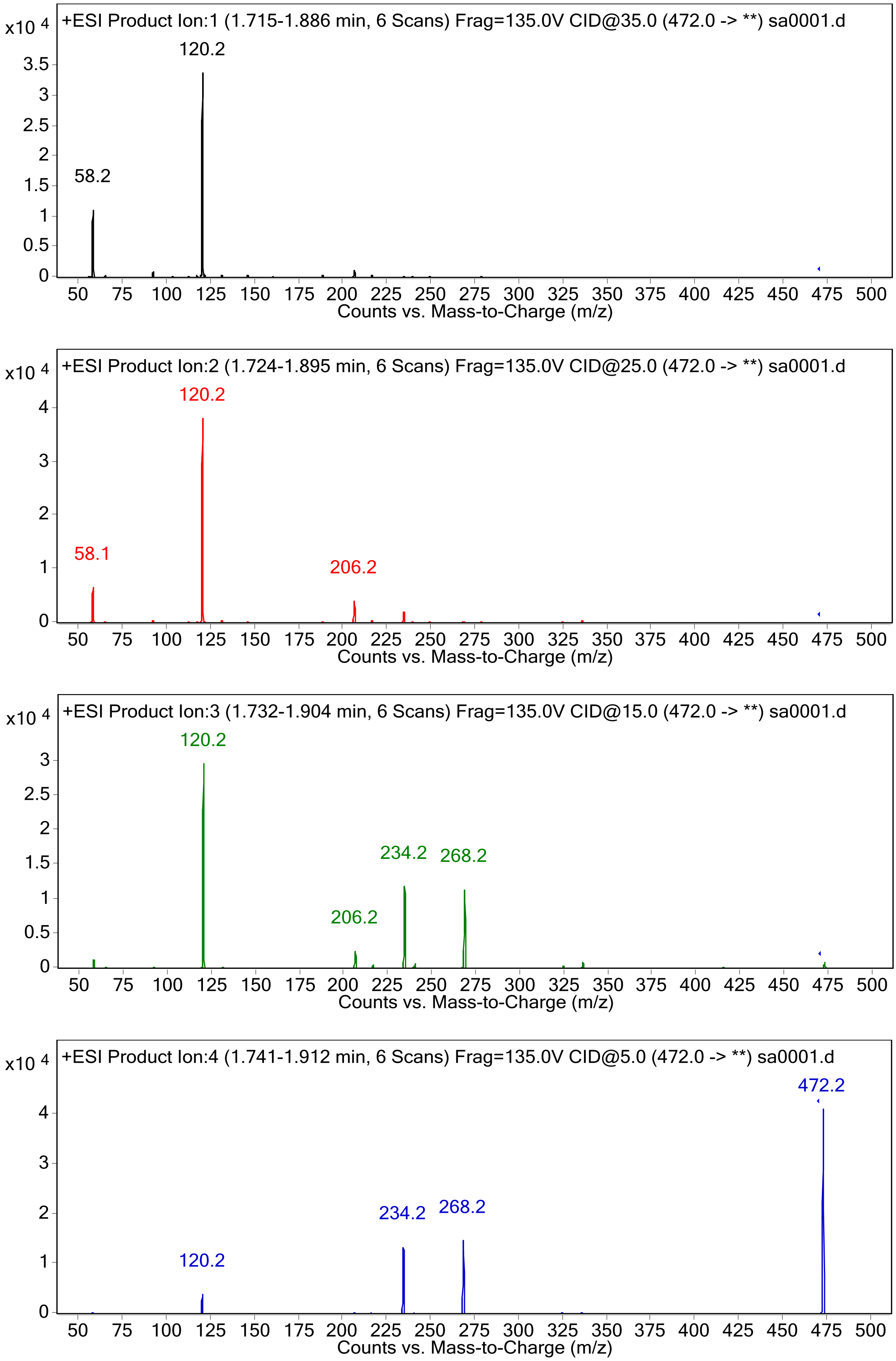

Supplement: Supplementary File 1 — Supplementary Information (PDF, 1569 KB) [file marinedrugs-11-04761-s001.pdf]
